# Measuring Visual Function Using the MultiQuity System: Comparison with an Established Device

**DOI:** 10.1155/2014/180317

**Published:** 2014-12-16

**Authors:** Patrycja Smolarek-Kasprzak, John M. Nolan, Stephen Beatty, Jessica Dennison, Kwadwo Owusu Akuffo, Robert Kuchling, Jim Stack, Graham O'Regan

**Affiliations:** ^1^Macular Pigment Research Group, Department of Chemical & Life Sciences, Waterford Institute of Technology, Waterford, Ireland; ^2^Institute of Vision Research, Whitfield Clinic, Waterford, Ireland; ^3^Macular Pigment Research Group, Vision Research Centre, Carriganore House, Waterford Institute of Technology, West Campus, Carriganore, Waterford, Ireland; ^4^Sightrisk Ltd., Carriganore House, Waterford Institute of Technology, West Campus, Carriganore, Waterford, Ireland

## Abstract

*Purpose*. To compare measures of visual acuity (VA) and contrast sensitivity (CS) from the Thompson Xpert 2000 and MultiQuity (MiQ) devices. *Methods*. Corrected distance VA (CDVA) and CS were measured in the right eye of 73 subjects, on an established system (Thompson Xpert) and a novel system (MiQ 720). Regression was used to convert MiQ scores into the Thompson scale. Agreement between the converted MiQ and Thompson scores was investigated using standard agreement indices. Test-retest variability for both devices was also investigated, for a separate sample of 24 subjects. *Results*. For CDVA, agreement was strong between the MiQ and Thomson devices (accuracy = 0.993, precision = 0.889, CCC = 0.883). For CS, agreement was also strong (accuracy = 0.996, precision = 0.911, CCC = 0.907). Agreement was unaffected by demographic variables or by presence/absence of ocular pathology. Test-retest agreement indices for both devices were excellent: in the range 0.88–0.96 for CDVA and in the range 0.90–0.98 for CS. *Conclusion*. MiQ measurements exhibit strong agreement with corresponding Thomson measurements, and test-retest results are good for both devices. Agreement between the two devices is unaffected by age or ocular pathology.

## 1. Introduction

The visual world is made up of high-definition (HD) environments. In the 21st century, everyday lives are becoming increasingly dependent on HD visual function, not just for activities such as mobility [1], reading performance [2], motor vehicle driving [3], and facial recognition [4], but also for our interactions with and accuracy in performing tasks using HD display screen technologies [5]. Navigation through this HD era requires good visual acuity, and the ability to detect objects at low contrast is also becoming increasingly important for these activities of daily living. However, despite the advances in visual display technologies, there has been a lag in innovation in terms of how visual function can be reliably and accurately assessed in this more visually demanding world, and there is a question as to whether the traditional techniques are appropriate for the real world of today.

For visual acuity (VA), the Snellen chart was first introduced in 1862 by Herman Snellen and the logMAR chart in 1976 [6]. However, a major limitation of VA measurement techniques is that they measure minimum angle of visual resolution using high-contrast targets only, whereas the real world is made up of objects across a range of spatial frequencies and contrast. It has been demonstrated that contrast sensitivity (CS) is more closely related to levels of disability and health-related quality of life related to vision in patients with ocular disease [7–9] and as such has been recommended for use in the clinical setting, particularly in low vision clinics [10], and also in the detection and monitoring of eye diseases such as glaucoma [11], cataract [12], diabetic retinopathy [13], and optic neuritis [14], as well as in the postoperative assessment of patients having undergone laser refractive procedures [15]. However, and in spite of these observations, CS is still not widely measured in the clinical setting [16], possibly reflecting difficulties in incorporating these measures into a busy clinical practice and a lack of appreciation of its value amongst eye care professionals.

Measures of CS can be classified as periodic pattern (sine-grating) [17] and letter-based (nonperiodic) [18] techniques. The letter-based Pelli-Robson CS chart represents a popular method of measuring CS [19]. However, the Pelli-Robson chart does have several limitations, and these are attributable to it being comprised of eight spatial frequencies (each frequency consisting of two triplets) and because it can prove difficult to ensure uniform illumination across the chart [20]. The MARS letter CS chart represents a redesign of the Pelli-Robson chart, and this uses the same technique, but with a more convenient hand-held (23 × 36 cm) chart and a contrast range from 0.04 to 1.92 log units [21]. Studies looking at the agreement of the MARS chart with the Pelli-Robson chart show variable results. Subjects with poorer CS have been found to score better on the MARS chart than on Pelli-Robson testing and to exhibit better repeatability of readings [22, 23]. Exceptions were subjects with normal vision, who have been reported to exhibit subtly better repeatability with the Pelli-Robson chart [22]. Number-based charts have also been developed and their validity has also been assessed [24], but they are still not widely used.

In more recent times, there has been an attempt to shift away from the traditional and somewhat coarser and cumbersome systems, in favour of computer-based applications to assess visual function. The Thompson Test Chart Pro 2000 CS test displays optotypes on a computer monitor and is similar to the Pelli-Robson test in the method it employs.

To our knowledge, there have been no published concordance studies between measures of visual function recorded on older LCD (liquid crystal display) and systems using these more recently introduced screen technologies.

We live in a HD world, and yet most eye care professionals restrict measures of visual function to systems that are insufficiently sensitive to represent a reflection of everyday visual demands and satisfaction. The MultiQuity (MiQ) test chart system (Sightrisk Ltd., Waterford, Ireland) represents a suite of measures of visual function that has been developed for use on both HD PDA (high definition portable digital assistant) and television-based monitor platforms. It has been designed to be used in everyday clinical practice, and its functions include measures of the following psychophysical parameters: VA and CS. The MiQ VA Test Suite has over 720 letter size increments, thereby offering advantages over traditional systems, not just in terms of avoiding crowding phenomena but also in terms of sensitivity. The high degree of acuity refinement also provides for improved sensitivity at the end point of refraction because the patient is closer to the threshold of letter recognition.

The aim of this study is to assess concordance between the measures of VA and CS recorded on the novel MiQ test chart versus the Thompson Test Chart 2000 Xpert. The Thompson Test Chart 2000 Xpert was chosen as it is the only computer-based system to have been compared with MARS and Pelli-Robson tests [20].

## 2. Methods

### 2.1. Setting and Subjects

This study was conducted at the Vision Research Centre, Waterford Institute of Technology, Waterford, Ireland. For the main analysis, a total of 73 subjects were recruited into the study. Inclusion criteria included any subjects older than 18 years who were willing to participate in the study. There were no exclusion criteria for subjects recruited into this study. Subjects were recruited by word of mouth, social media, and existing subject databases at the Macular Pigment Research Group (MPRG); we specifically included some subjects with age-related macular degeneration (AMD) and cataract (see Section 2.6). Ethical approval was granted from the local Waterford South East (of Ireland) Region Ethics Committee prior to the study commencing.

In all cases, the right eye was used as the study eye. Subjects wore an occlusive eye patch over the nontested (left) eye. Testing was performed by a single researcher (Jessica Dennison). The room lights were on for all tests. In order to avoid bias with respect to the test conducted first, subjects were randomized to either the MiQ or Thompson Test Chart Xpert 2000 at the beginning of the study. An additional 24 subjects were recruited for test-retest assessment. The second test for the test-retest assessment was performed the next day for the purpose of this analysis.

### 2.2. Acuity Testing (Thomson)

The method for assessing Thompson VA has been described in another study [20]. A computer-generated LogMAR test chart was used (Thomson Test Chart 2000 Xpert displayed on an HP monitor LV916AA2211 (resolution 1920 × 1080, luminance 250 cd/m^2^, dynamic contrast ratio 3,000,000 : 1)), which exceeds the minimum Thomson specifications (Thompson Software Solutions, Hertfordshire, UK). Corrected distance VA (CDVA) was measured at a viewing distance of 4 m (direct). The Sloan Early Treatment Diabetic Retinopathy Study (ETDRS) letterset was used for this test. The patient was instructed to read the line of letters, starting with the largest size and continuing downwards until a line was reached which was incompletely or incorrectly read. The letters of the line were randomized three times using the testing software's randomization function and an average of three scores was taken. CDVA was recorded as visual acuity rating (VAR).

### 2.3. Acuity (MiQ 720)

CDVA was also measured using the MiQ 720 (part of the MiQ test Suite), a computerized test chart. The test is remotely controlled by the researcher using an Apple iPad. The iPad display is mirrored on a HD LED screen or monitor.

In this study, an LG TV was used (HD LED, resolution 1920 × 1080, contrast ratio 9,000,000, luminance 250 cd/m^2^). The viewing distance used was 4.05 m direct. A dedicated letterset and font type are used for this test, comprising the letters C, O, U, V, X, and Z. This letterset is designed to minimize the well-known problems associated with interletter recognition for both acuity and contrast testing [25–27]. The use of a three-letter display provides further control over the interletter recognition variability by reducing the size of the letter pool, whilst still enabling randomized letter generation.

**Figure 1:**
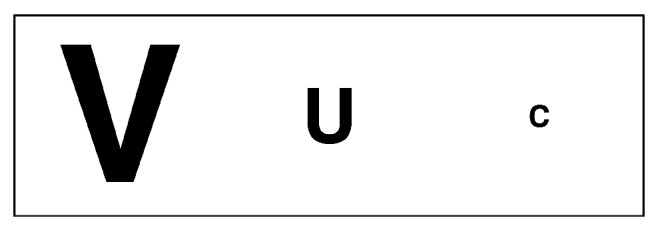
An example of the algorithmically generated letter triplet for testing VA. VA: visual acuity.

Three randomized letters are presented using a computerized repetitive refinement algorithm. The first display comprises the very largest and the very smallest of the available letters within the test and an intermediate size. The smallest letter correctly read aloud by the subject is selected by the examiner, by tapping the appropriate letter on the iPad display, and the next algorithmically generated letter triplet is displayed. An example is shown in Figure 1. The process is repeated until the termination line is reached and a results' page is displayed.

The algorithm is designed not only to considerably speed up the journey to the end point but also to equally speed up and increase the sensitivity of the testing procedure. This is enabled by allowing refinement at any level of the algorithm, as there will always be letters displayed above, below, and close to acuity threshold. As the repetitive refinement algorithm is self-refining, no repeat testing or averaging of results is necessary, and results are immediately displayed without any requirement for the complex and slow adjustment of the result inherent in the logMAR scoring system.

Results are generated in MiQ units (across a range from 0 to 100 in steps of one decimal place). The results' page also displays the mathematical conversion to VAR, logMAR, and Snellen.

### 2.4. Contrast Sensitivity (Thomson)

Letter CS was assessed using the computerized LogMAR ETDRS test chart contrast test (Test Chart 2000 Xpert; Thomson Software Solutions). The Sloan ETDRS letterset was displayed at the 6/24 (Snellen) size (approximating to 6 cycles per degree cpd) and subjects were asked to read the letters aloud whilst fixating on the chart at a viewing distance of 4 m (direct). The letterset was randomized during the test at each change of contrast. The percentage contrast of the letters was decreased to 0.15 logCS steps until the lowest contrast value for which subjects saw at least three letters was reached. Each letter has a nominal logCS value of 0.03. Missed and incorrectly read letters at any contrast level were noted. The resultant logCS value for the subject was calculated by adding any extra letter(s) and/or subtracting missed letters from the best logCS value corresponding to the lowest percentage contrast. This protocol is the Pelli-Robson scoring system.

### 2.5. Contrast Sensitivity (MiQ)

The MiQ Contrast 256 test was performed on the LG TV described above. The viewing distance used was 4.05 m direct. A dedicated letterset and font type are used for this test, comprising the letters A, C, E, H, N, R, S, and Z. Three randomized letters are presented using a computerized repetitive refinement algorithm. The letters are displayed in negative contrast (light on dark) to minimize glare effects. The first display presents the first letter at the highest contrast of the available contrasts within the test (approximately 0.1 logCS), the third letter at a contrast below the threshold of visibility, and the second letter (central) at an intermediate contrast.

**Figure 2:**
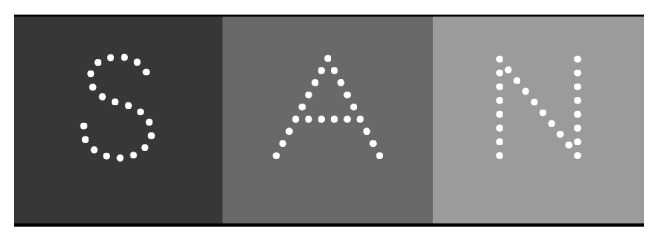
An example of the algorithmically generated letter triplet for testing CS. CS: contrast sensitivity.

The lowest contrast letter correctly read aloud by the subject is selected by the researcher who taps on the iPad display, and the next algorithmically generated letter triplet is displayed (Figure 2). The process is repeated until the end point is reached.

The contrast result is displayed in MiQ contrast units. Results are also displayed in the mathematical conversion to logCS and Weber contrast.

### 2.6. Statistical Methods

Statistical analyses were conducted using SPSS 19.0 (SPSS, Inc., Chicago, Ilinois, USA) and the statistical programming language R (R Foundation for Statistical Computing).

It is evident from previous studies that a sample of size 50 often has acceptable power properties for this type of agreement study [28, 29]. However, we increased the sample size to 73 in our study and deliberately included subjects with conditions such as AMD (12 subjects) and cataract (8 subjects) in order to ensure a wide range of VA and CS scores for comparison purposes. Volunteers for this study were not screened for other ocular pathologies.

Due to the different scales of measurement from the two devices, ordinary least squares regression (OLS) was used to convert CS scores from the MiQ scale into the Thomson logCS scale. Agreement between these converted logCS estimates and actual Thomson logCS was then investigated using standard agreement indices: accuracy coefficient, precision coefficient, and the concordance correlation coefficient (CCC); these indices are presented and explained in the appendix below. The regression transformation, to the Thompson scale, makes the means of the two variables equal, and the accuracy component of agreement is affected by this, but we still elected to use the CCC (of which the accuracy coefficient is a component) because we regard it as the best single measure of agreement. The paired *t*-test for bias (often applied in agreement studies) becomes redundant, however, because the means are guaranteed to be equal. Lower confidence limits for concordance, precision, and accuracy coefficients were obtained from R code supplied with Lin et al. [28]. The possible effect on agreement of age, gender, AMD, and cataract was investigated, using a general linear model. Agreement was also investigated graphically; here, we preferred to use an ordinary scatterplot of the two variables being compared (with line *y* = *x* super-imposed) rather than the more usual Bland-Altman plots with limits of agreement displayed. In our experience, these simpler plots are more effective in visually assessing agreement, because they are easier to understand and also because they graphically depict what is being measured by the CCC.

We recruited a separate sample of 24 volunteers (without screening for ocular pathologies) for purposes of test-retest statistical analysis. The same statistical and graphical methods were applied to investigate agreement between Thomson and MiQ devices and to investigate test-retest variability of each device. In test-retest analyses, however, each device is compared with itself, and there is no need to transform from the MiQ scale to the Thompson scale.

## 3. Results

Study subjects (*n* = 73) ranged in age from 21 to 80 years, and mean (±st. deviation) was 46.6 (±17.3) years. Forty-five subjects (61.6%) were males.

### 3.1. Agreement between Devices: CDVA

**Figure 3:**
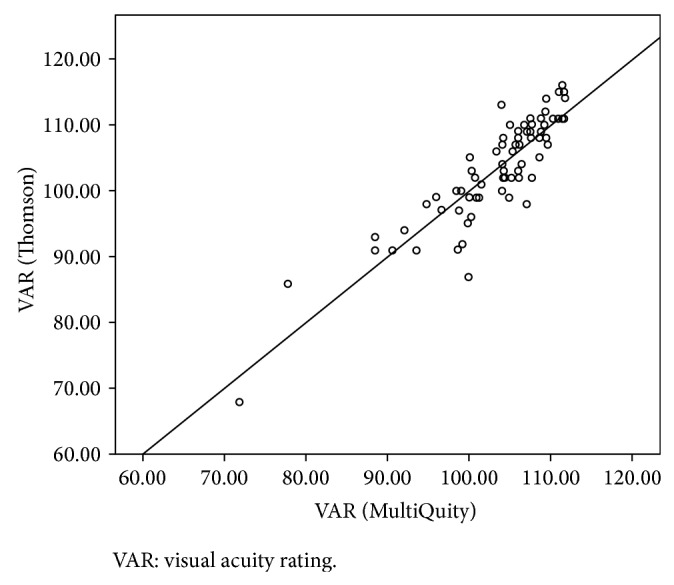
Agreement between visual acuity rating from the Thomson device and estimated visual acuity rating from the MiQ device. VAR: visual acuity rating.

Agreement was strong between the two devices for CDVA. After conversion of MiQ VAR scores to Thomson VAR scores, via regression, we obtained the precision, accuracy, and concordance indices reported in Table 1. The scatterplot of Thomson VAR and MiQ estimate of Thomson VAR are presented in Figure 3. The line of equality (*y* = *x*) has been overlaid on the plot, and the closeness of the scatter of points to this line, through the whole range of VAR values, demonstrates strong concordance between the two devices.

### 3.2. Agreement between Devices: CS

**Figure 4:**
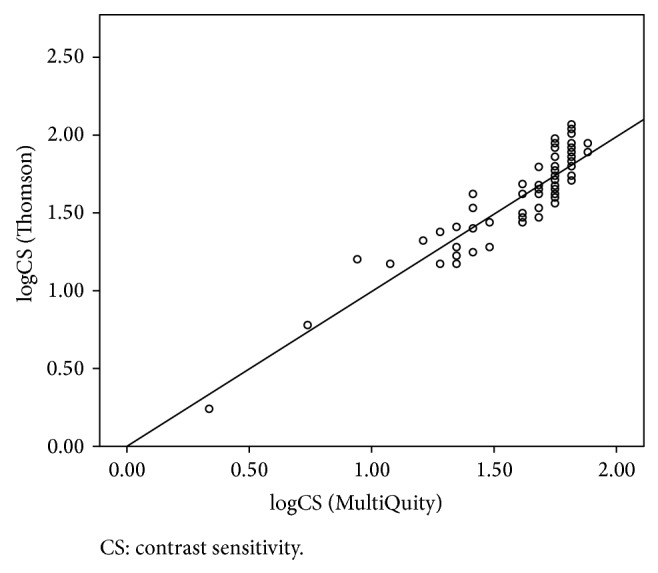
Agreement between log contrast sensitivity from the Thomson device and estimated logCS from the MiQ device. CS: contrast sensitivity.

Agreement was strong between the two devices for CS. After conversion of MiQ scores to Thomson logCS scores, via regression, we obtained the precision, accuracy, and concordance indices reported in Table 1. The scatterplot of Thomson logCS and MiQ estimate of Thomson logCS are presented in Figure 4. The line of equality (*y* = *x*) has been overlaid on the plot, and the closeness of the scatter of points to this line, through the whole range of logCS values, demonstrates strong concordance between the two devices.

### 3.3. Effect on Agreement of Disability or Demographic Variables

#### 3.3.1. CS

Agreement of CS scores from the two devices was unassociated with any of these study variables: age; sex; AMD status; cataract status (general linear model, *P* > 0.05 for all).

#### 3.3.2. CDVA

Agreement of CDVA scores from the two devices was unassociated with age, AMD status, or cataract status (general linear model, *P* > 0.05 for all). Agreement was affected by sex (*P* = 0.014), with Thomson yielding, on average, slightly higher VAR for males and slightly lower VAR for females, but the actual differences in both cases were less than one in magnitude and not clinically meaningful.

### 3.4. Repeatability of Measurements

There was good agreement between test and retest measures of both CDVA and CS from both the Thompson and the MiQ devices (Table 2).

## 4. Discussion

In this concordance study of the Thompson Test Chart 2000 Xpert versus MiQ, readings of measures of CDVA and CS recorded on the two devices were found to be concordant, and test-retest reliability was good for both devices.

Assessment of CS is useful in the screening and diagnosis of ocular disease, assessing, and monitoring visual function and for the prediction of vision-related ability [7–15]. It has also been reported that CS for low and medium spatial frequencies may be reduced in patients with ocular pathologies, even when CDVA is normal [30, 31]. However, despite the relevance of CS assessment, currently available test systems are expensive, difficult to set up and calibrate, and often prohibitively time-consuming for the clinical setting [16].

The development of computer-based systems to assess parameters of visual function has represented an important paradigm shift in recent times. Computer-based displays offer some advantages over traditional systems for assessing VA and CS. First, ease of portability lends them for use in domiciliary visits. Also, for VA testing, the sequence of presenting optotypes can be randomized, thereby negating any contribution that memorization may make to the values recorded. Further, changes can be made in stimulus parameters, such as spacing arrangement, contrast, optotype size, exposure time, and luminance. One of the most commonly used computer-based systems is the Thompson Test Chart 2000 [20].

Until recently, it was difficult to obtain sufficient luminance on electronic LCD screens. Pixel structure also creates limitations—for reasonable shape fidelity, letters need to be ≥10 pixels in height, and the need for more pixels may be more evident for Landolt ring and tumbling E targets with their more regular structures [32]. However, modern display technology offers high resolution and pixel density that overcomes some of the difficulties observed in the past that were related to poor resolution and luminance. Some modern displays have a pixel density so high that the human eye would be unable to appreciate further enhancement of pixel density at a typical viewing distance [33]. However, until now, existing computer-based systems for visual testing have failed to take full advantage of the opportunities that modern screen technologies represent, as they have essentially replicated the original wall charts of Snellen and Bailey-Lovie, reflected in the traditional and narrow range of acuity levels being displayed in wide steps.

The MiQ system uses a computer algorithm to generate results of CDVA and CS over a range that is close to being continuous, with a range approaching 1,000 levels. Such a fine grading scale, when used with a modern flat screen display, allows for a potentially greater degree of accuracy.

Valid and repeatable measures of CDVA and CS are essential for research and clinical settings. Presently, the logMAR and Pelli-Robson charts are considered to be the gold standard tests for assessing CDVA and CS, respectively [34]. However, even these tests exhibit poor test-retest repeatability in the clinical setting, and there is an increasing awareness that chart design affects measurements [22, 23]. Given the limitations of the tools at hand, there remains an unmet need for better (more reliable, accurate, and practical) systems of visual function testing for eye care professionals.

Computerized vision test systems are becoming more innovative; as the quality of displays and functionality of applications continue to improve, it is likely that eye care professionals will be empowered to accurately and reproducibly record more subtle measures and changes in such measures of vision that were hitherto imperceptible with traditional systems. Given the emerging technologies and falling prices of modern flat screen displays, computerized test systems represent a cost-effective alternative to conventional charts and projectors with uniform luminance and stability of testing conditions over time, with the further benefit in terms of ease of portability.

It is always challenging to introduce new systems into clinical practice, and the first step is to validate and to assess test-retest variability of any novel technology. In this concordance study of the Thompson Test Chart 2000 Xpert versus MiQ, measures of CDVA and CS on the two devices were found to be concordant, and test-retest consistency was also high for both devices. However, we recommend that further research using this novel technology investigates concordance across different populations (e.g., patients with cataract, AMD, glaucoma, etc.), as this will be important to confirm agreement in these populations of interest.

The novel MiQ test system offers several potential advantages over other systems, as the number of increments of visual function tested and recorded is far greater than alternative techniques, thus facilitating finer measures of visual function.

## Notes

Current displays are vastly better than previously available; however, they still have limitations. HD LEDs have higher luminance and a lower contrast range than plasma displays (which are currently unavailable other than on expensive and very large TV screens). The higher luminosity of LED screens necessitates the need for close control over calibration. This is a direct consequence of the Weber-Fechner law, which is also the explanation for the poor results obtained when using the old CRT displays.

## Figures and Tables

**Table 1:** Agreement indices for measurement of CDVA and CS by Thomson and MiQ devices.

Measure	CCC	Precision	Accuracy
CDVA	0.883 (0.83)	0.889 (0.84)	0.993 (0.97)
CS	0.907 (0.87)	0.911 (0.87)	0.996 (0.98)

CCC: concordance correlation coefficient; CDVA: corrected distance visual acuity; CS: contrast sensitivity; MiQ: MultiQuity.

For each coefficient, the 95% lower confidence limit is shown in brackets (based on *n* = 73 subjects).

**Table 2:** Agreement indices for test-retest of CDVA and CS by Thomson and MiQ devices.

Measure	CCC	Precision	Accuracy
CDVA Thomson	0.896 (0.847)	0.961 (0.921)	0.933 (0.891)
CDVA MiQ	0.885 (0.800)	0.922 (0.847)	0.959 (0.896)
CS Thomson	0.931 (0.877)	0.960 (0.921)	0.970 (0.928)
CS MiQ	0.903 (0.823)	0.919 (0.840)	0.983 (0.927)

CCC: concordance correlation coefficient; CDVA: corrected distance visual acuity; CS: contrast sensitivity; MiQ: MultiQuity.

For each coefficient, the 95% lower confidence limit is shown in brackets (based on *n* = 24 subjects).

## Appendix

Notation is as follows: variables X and Y , means Mean( X ) and Mean( Y ), standard deviations SD( X ) and SD( Y ), and covariance Cov (X,Y) . Consider

(A.1)1Precision=Cov(X,Y)SDX*SD(Y).

Precision is the ordinary Pearson correlation coefficient and measures the degree of scatter in the (X,Y) plot around the best-fitting regression line. Consider

(A.2)2Accuracy=2w+1/w+v2,

where $w=\text{SD}\left(X\right)/\text{SD}(Y)$ and v = (Mean( X ) − Mean( Y ))/ $\sqrt{\text{SD}\left(X\right)*\text{SD}(Y)}$ .

Accuracy will be close to 1 if the two means are close in value and the two standard deviations are close in value. Consider

(A.3)3Concordance=Precision*Accuracy.

Concordance will be close to one if precision and accuracy are both close to 1.

## Abbreviations

HD: High definition
HD LED: The current state-of-the-art monitor display
LED: Light emitting diode
MiQ: MultiQuity.
